# Conventional physical examination extended by bedside ultrasound: a new paradigm in nephrological practice

**DOI:** 10.1590/2175-8239-JBN-2020-0069

**Published:** 2020-07-08

**Authors:** Ana Cláudia da Silva, Fabiana Oliveira Bastos Bonato, Marcus Gomes Bastos

**Affiliations:** 1 Universidade Federal de Juiz de Fora, Juiz de Fora, MG, Brasil.

**Keywords:** Amyloidosis, Ultrasonography, Nephrotic Syndrome, Amiloidose, Ultrassonografia, Síndrome Nefrótica

## Abstract

Point-of-Care Ultrasound (POCUS) has been gaining momentum as an extension to physical examination in several specialties. In nephrology, POCUS has generally been used in a restricted way in urinary tract evaluation. We report the case of a patient with nephrotic syndrome secondary to amyloidosis, previously diagnosed by renal biopsy, who was oligosymptomatic when seen the an outpatient clinic, where the POCUS, focused on the heart, lung and abdomen, revealed anasarca, pulmonary congestion and cardiac changes suggestive of cardiac amyloidosis. After evaluation by the cardiology and hematology services, the diagnosis of AL amyloidosis with cardiac involvement was confirmed. This case emphasizes the importance of extending the physical examination using POCUS, which, ideally, should not be restricted to the urinary tract.

## RESUMO

A ultrassonografia Point of Care (POCUS) vem ganhando momentum como uma extensão ao exame físico em várias especialidades. Na nefrologia, a POCUS tem sido geralmente utilizada de forma restrita na avaliação do trato urinário. Relatamos o caso de uma paciente com síndrome nefrótica secundária à amiloidose previamente diagnosticada por biópsia renal, que se apresentava oligossintomática quando atendida em ambulatório, onde a POCUS, com foco no coração, pulmão e abdômen, revelou anasarca, congestão pulmonar e alterações cardíacas sugestivas de amiloidose cardíaca. Após avaliação pelos serviços de cardiologia e hematologia, foi confirmado o diagnóstico de amiloidose AL com envolvimento cardíaco. Esse caso enfatiza a importância da extensão do exame físico pela POCUS, que, idealmente, não deve se restringir ao trato urinário.

## INTRODUCTION

Historically, physical examination is a fundamental step in the patient’s medical evaluation and, consequently, clinical decision-making. There is a recent proposal to add insonation^1^ to inspection, palpation, percussion and auscultation, which are the traditional pillars of physical examination. Ultrasonography, used by the non-image specialist at the time of the physical examination, as an extension of it, is called “point of care” ultrasound (POCUS)^2^. Almost all medical specialties are using POCUS today and, more recently, it has gained momentum in nephrology.^3^^,^ 4

The perception that ultrasound can improve physical examination was more evident in the 1990s, particularly from the needs of American emergency physicians. POCUS provides additional clinical information to decision making, motivating several American and Canadian medical schools to include it in medical undergraduate education.^5^^,^ 6 In Brazil, the POCUS teaching initiative in undergraduate medicine appeared in the middle of 2013.^7^ The initiative to train nephrologists took place in 2014, at the Brazilian Congress of Nephrology. When tested in a group of residents, the evaluations showed the development of skills in obtaining images and performing nephrological procedures.^8^

Ultrasonography should not be used alone in the diagnosis of a certain pathology, whether renal or in another organ. The correlation between clinical history, physical examination and POCUS is fundamental in the diagnostic process. Below, we present a clinical case of a woman with nephrotic syndrome due to renal amyloidosis, with the objective of illustrating the beneficial results of this integration of knowledge in nephrological practice.

## STRUCTURED CASE PRESENTATION

D.M.S.V., 53 years old, woman, black, born in and resident of Além Paraíba, MG, was diagnosed with nephrotic syndrome secondary to renal amyloidosis (confirmed by renal biopsy). The patient was referred to the outpatient glomerulopathies service of the University Hospital of the Federal University of Juiz de Fora for therapeutic evaluation.

During the consultation, the patient had no complaints, other than edema in both legs. Upon physical examination, she had blood pressure of 150 x 100 mmHg, crackles in the lungs upon auscultation, semi-globose abdomen and edema of the lower limbs (3/+4). The patient had blood tests carried out in February 2018, showing: creatinine = 1.7 mg/dL; estimated glomerular filtration rate (eGFR) = 34 mL; min/mg/dL; and in urine: proteinurua 1.73m^2^; albumin = 2.2 mg/dL. Moreover, in urinalysis: proteinuria of 4.5 g/24h. Tests carried out in July of the same year, at the time of the renal biopsy, showed serum creatinine of 1.24 mg/dL; eGFR = 50 mL/min/1.73m^2^ and proteinuria of 2.7g/24h. At the clinic of origin, she was treated with prednisone 1 mg/kg; furosemide 40 mg/day; spironolactone 25 mg/day; simvastatin 40 mg/day, and clopidogrel 75 mg/day.

**Figure 1:**
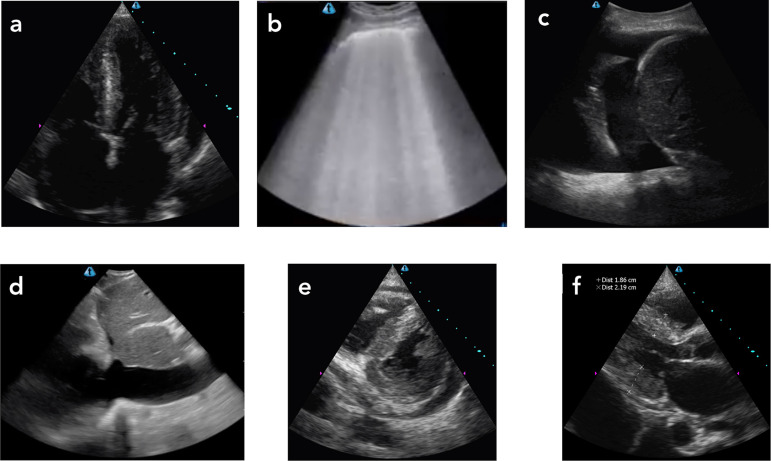
Ultrasound images obtained during the nephrological consultation: a. Heart with large atrial enlargements, increased thickness of the interventricular septum and pericardial effusion seen in the apical cardiac window; b. B lines seen in both lungs; ç. Pleural effusion on the right, evidenced by the replacement of the mirror image of the liver by an anechoic image (liquid) and visualization of vertebral bodies above the diaphragm; d. “Plethoric” inferior vena cava without changing its diameter during the respiratory cycle. Cardiac image obtained through the paraesternal short axis window, showing interventricular septum paraesternal short axis window, with the left ventricle in a "D" shape due to the increased pressure and volume of the right ventricle, and pericardial effusion; f. Increased thickness of the interventricular septum and the left ventricular posterior wall in diastole and a large left atrium increase.

**Figure 2:**
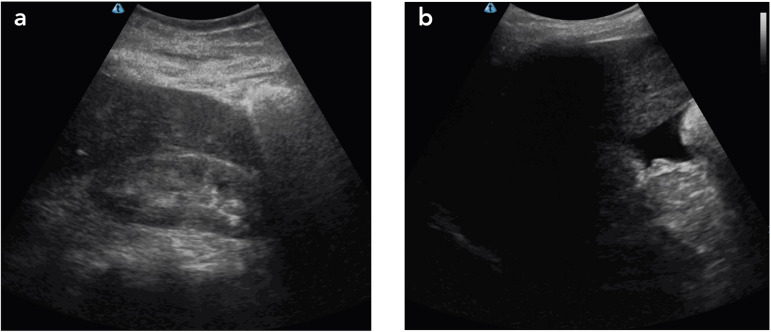
a. Right kidney with hyperechoic cortex and loss of corticomedullary differentiation, compatible with the chronic glomerulopathy due to amyloid deposit presented by the patient; B. Anechoic image with acute angles, typical of free intraperitoneal fluid, in this case ascites, seen at the level of the right kidney’s lower pole.

The nephrology team performed the POCUS examination at the bedside as part and extension of the physical examination, which revealed signs of hypervolemia, bilateral pleural effusion, pericardial effusion, ascites, and B lines in more than two examined areas of both lungs (Figures 1 and 2). Besides, it was observed increased thickness of both the interventricular septum (granular in appearance) and the left ventricular posterior wall, an enlargement in the atria, and a reduction in the amplitude of the basal displacement of the septal annulus of the mitral valve during diastole. Also found were a limitation of the movement of the mitral valve leaflet relatively to the interventricular septum, mitral and tricuspid regurgitations, enlargement of the right ventricle, with the rectification of the interventricular septum (called “D” sign), and plethoric inferior vena cava which does not change its diameter with the respiratory cycle. The kidneys were hyperechogenic, with some loss of corticomedullary differentiation (Figure 2).

Based on the described findings, we formulated the diagnostic hypothesis of amyloidosis with cardiac and renal involvement. The patient was admitted for compensation of the renal and cardiac conditions and cardiological and hematological evaluations.

Upon admission, the electrocardiogram showed sinus tachycardia (120 bpm), left atrial overload, and low voltage QRS and left axis deviation, in addition to secondary alteration of anterolateral ventricular repolarization. A cardiologist with expertise in echocardiography confirmed the fidings described by the nephrologists. Finally, Cardiac Nuclear Magnetic Resonance imaging (MRI) evidenced the absence of detection of the null myocardial inversion time following delayed enhancement (suggestive of cardiac amyloidosis), ventricular hypertrophy and thickening of the interatrial and interventricular septa. 

Cardiac MRI, plus protein electrophoresis, serum and urine immunofixation, and bone marrow biopsy with immunohistochemistry, enabled the diagnosis of AL amyloidosis with renal and cardiac involvement.

## DISCUSSION

The case presented confirms that POCUS can assist the nephrologist in the diagnosis of cardiac amyloidosis when one extends the physical examination with ultrasonography in a patient with nephrotic syndrome due to renal amyloidosis. In similar cases, in most services, the patient's ultrasound assessment would be conducted by an imaging specialist after the assistant physician's request or, in a minority of cases, performed by the nephrologist himself, however with a restricted approach to the urinary tract.^9^ In this sense, recent publications have proposed that as an extension of the nephrological physical examination, a more holistic assessment of POCUS is possible and desirable, thus, allowing the nephrological practice to reach a higher level.

Functional amyloid fibrils are protein polymers comprising identical monomer units that play a beneficial role in various physiological processes. Amyloidosis is a clinical disorder caused by the intra and/or extracellular deposition of pathogenic amyloids fibrils - most of which are aggregates of misfolded proteins - that alters normal tissue functionality.^10^ Amyloidosis is currently classified chemically. The types of amyloidosis are referred to with a capital A (for amyloid) followed by an abbreviation for the altered fibril protein. Amyloidosis caused by abnormal deposition of fragments of immunoglobulin light chains (abbreviated L) is now called AL amyloidosis. Cases of amyloidosis due to abnormal deposition of trnasport protein transthyretin (TTR) are collectivelly termed ATTR.^11^ In the case presented, the hematological diagnosis was AL amyloidosis, with abnormal deposition of amyloid fibrils in the kidneys and heart.

The kidneys and the heart are the organs most frequently affected in AL amyloidosis, although all others can be affected, except the brain. The main renal manifestations of AL amyloidosis are nephrotic syndrome, chronic kidney disease, and edema, all present in the case under discussion at the time of consultation. However, the use of POCUS enabled the identification of clinical changes not detected by physical examination. Ascites, pleural, effusion and pericardial, effusion (anasarca) showed that the patient’s water retention was more accentuated than previously suggested only by lower limb edema. The finding of renal amyloidosis confirmed by renal biopsy and the observations of diastolic dysfunction, septal and left ventricular posterior wall thickening, detected by focused cardiac ultrasound,^12^ raised the suspicion of cardiac amyloidosis,^13^ which only occurred because the performance of POCUS in the consultation was not restricted to the urinary tract.

Cardiac dysfunction of AL amyloidosis can result from amyloid deposits with generalized breakdown in tissue architecture and proteotoxicity of light chains, with consequent necrosis of cardiomyocytes and interstitial fibrosis.^14^ Clinically, the disease is most frequently expressed by heart failure with preserved ejection fraction, thickened ventricular walls on transthoracic echocardiography and low voltage on the electrocardiogram, dyspnea at rest and exercise, fatigue, hypotension or syncope and peripheral edema. Cardiac AL amyloidosis is an aggressive disease and its diagnosis is a key determinant of patient survival, usually very short.^15^

Morphologically, AL amyloidosis is indistinguishable from APTT on echocardiography.^16^ The main characteristics of cardiac amyloidosis result from diastolic abnormalities due to increased thickness of the interventricular septum and ventricular wall by amyloid infiltration. In the case presented, the assessment of diastolic dysfunction was qualitative, based on an increase in the left atrium diameter and a reduction in the amplitude of the basal displacement of the septal annulus of mitral valve during diastole.^12^^,^ 17 The sign of the letter D, seen in the parasternal short axis window and the tricuspid regurgitation suggest increased pressure and volume in the right ventricule ,^18^ and explain the finding of the plethoric inferior vena cava, without varying its diameter with the respiratory cycle.^19^

At the time of consultation, the ratio of E and e’ waves, obtained by pulsed transmitral Doppler and tissue Doppler, respectively, was not calculated to determine the left ventricular end-diastolic pressure. However, it was evident that the patient had B lines diffusely distributed in both lungs. B lines are lung artifacts seen as vertical lines, which originate at the visceral pleura, move with the respiration, erase the A lines (parallel and equidistant horizontal lines, which mean air in the lung), and and traverse the entire ultrasound screen vertically to the bottom of the screen.^20^ The finding of three or more B lines between two ribs, at two or more evaluation points in both lungs, in the right clinical context, should be considered a diagnosis of pulmonary edema, with sensitivity and specificity of 94% and 92%, respectively.^21^ Recently, it has been shown that B lines on pulmonary ultrasound correlates with increased left ventricular end-diastolic pressure.^22^

This case, like others previously published, points out the importance of adding POCUS in the evaluation of renal patients, to provide complementary information of great clinical value. This is more evident when the patient does not have exuberant symptoms, and when there is a discrepancy between the findings of the physical examination and the clinical status, with resulting diagnostic uncertainty.
